# A Comparison between Two Heterodyne Light Sources Using Different Electro-Optic Modulators for Optical Temperature Measurements at Visible Wavelengths

**DOI:** 10.3390/s101109609

**Published:** 2010-10-29

**Authors:** Ruey-Ching Twu, Yi-Huan Lee, Hong-Yao Hou

**Affiliations:** Department of Electro-Optical Engineering, Southern Taiwan University, 1, Nan-Tai St., Yung-Kang City, Tainan County 71005, Taiwan; E-Mails: staunchlee@hotmail.com (Y.-H.L.); hou18612@hotmail.com (H.-Y.H.)

**Keywords:** heterodyne interferometry, electro-optic modulator, optical temperature sensor

## Abstract

In this paper we have successfully demonstrated a *z*-propagating Zn-indiffused lithium niobate electro-optic modulator used for optical heterodyne interferometry. Compared to a commercial buck-type electro-optic modulator, the proposed waveguide-type modulator has a lower driving voltage and smaller phase variation while measuring visible wavelengths of 532 nm and 632.8 nm. We also demonstrate an optical temperature measurement system using a homemade modulator. The results show that the measurement sensitivities are almost the same values of 25 deg/°C for both the homemade and the buck-type modulators for a sensing light with a wavelength of 632.8 nm. Because photorefractive impacts are essential in the buck-type modulator at a wavelength of 532 nm, it is difficult to obtain reliable phase measurements, whereas the stable phase operation of the homemade one allows the measurement sensitivity to be improved up to 30 deg/°C with the best measurement resolution at about 0.07 °C for 532 nm.

## 1. Introduction

Optical phase measurements, based on the Mach-Zehnder and Michelson interferometers, are critical for various fields, including biosensing, rotation-angle, and absolute-distance measurements [1–5]. An optical heterodyne technique has been widely used for improving phase measurement precision in optical interferometers, consequently a stable heterodyne light source (HLS) is essential for measurement resolution. An HLS can be obtained by using a Zeeman laser with an intrinsic frequency difference between two orthogonally polarized lights [1,2]. However, its frequency difference is fixed. The optical frequency of the lights can be shifted by using an acousto-optic modulator (AOM) [3,4]. Unlike the Zeeman laser, with a fixed frequency difference, the AOM is flexible for modulating frequency differences. By using the optical interferometric setup [4], both the different frequency lights combine and produce heterodynic interference signals. In two-frequency heterodyne interferometry, the output interferometric signals include a sum-frequency and a difference-frequency. The envelope of the sum-frequency part is a sinusoidal-like waveform. Usually, an electronic low-pass filter can filter out the high frequency signals. The extracted low-frequency signals are sinusoidal with a beat frequency (difference-frequency between two different frequencies). One-frequency heterodyne interferometry uses an electro-optic modulator (EOM) to provide a phase delay between both orthogonal polarizations of the same wavelength light. The incident lights are further modulated as the HLS by applying a prompt saw-tooth voltage onto the EOM with a π-phase modulation depth. Because the incident light in the AOM-based two-frequency heterodyne interferometry will be separated spatially for different frequency modulations and combined again at a common path, it will need more optical and electronic components compared with EOM-based common-path heterodyne interferometry. In the typical dual-channel common-path heterodyne interferometer, the sensing phase information is embedded in the sinusoidal waveform of the sensing signal that can be measured by comparing the phase difference with another sinusoidal waveform of the reference signal through a lock-in amplifier (LIA) [5,6]. The phase shift between the two sinusoidal waveforms also can be decided by utilizing a phase-shift dependent Lissajous curve or an appropriate signal processing of the raw photodiode signals, as described in [7].

In the past decade, a commercial buck-type EOM (Model 4002, New Focus, Inc.) made with an Mg-doped lithium niobate crystal (5 mol% Mg:LN) has been used to modulate the HLS for various interferometric measurement setups [5,6]. To reduce the modulated voltages, the highest electro-optic coefficient of *r*_33_ is adopted by applying the electric field parallel to the optical axis (*c*-axis) of the LN crystals. Therefore, the propagation direction of the incident light is perpendicular to the *c*-axis of the LN crystals. Because the gap between electrodes in the commercial EOM is around 2 mm, the peak values of the applied voltages for a π-phase shift between both orthogonal polarizations are over one hundred volts at a wavelength of 632.8 nm. The photorefractive-induced phase variations are obvious when the light propagates perpendicularly to the *c*-axis of the LN crystals. This will limit the input light powers at visible wavelengths. The photorefractive-induced phase variations can be decreased dramatically by using the incident light propagating parallel to the *c*-axis of the LN crystals [8]. However, the smaller EO coefficient of *r*_22_ will cause higher input voltages for buck-type EOM phase modulations. To make a stable phase modulator with a low driving voltage, a *z*-propagating (parallel to the *c*-axis) Ti-indiffused (TI) LN waveguide phase modulator has been successfully proposed for the polarization scrambler in the near-infrared wavelengths [9]. However, an unstable conversion efficiency of a z-propagating TI polarization converter was observed due to the photorefractive phase drift at visible wavelengths [10]. Moreover, we compared the phase stabilities between the ZI phase modulator and the TI one at 632.8 nm, based on homodyne metrology [11]. The phase jumps of the TI phase modulator makes phase noise for the optical phase measurement systems unpredictable. This indicates that the *z*-propagating TI phase modulator is still difficult to use for optical metrology at visible wavelengths [10,11].

In this study, we have, for the first time, successfully demonstrated a waveguide-type Zn-indiffused LN electro-optic modulator (ZIEOM) to modulate the HLS being used in heterodyne interferometry. Comparisons of phase stabilities between the homemade ZIEOM and the EOM have been explored under different throughput powers at the same applied voltages with a frequency of 100 kHz. The experimental results show that the ZIEOM has the potential to generate a phase stable HLS at visible wavelengths with a low driving voltage. We have further compared the measurement performance by utilizing both the ZIEOM and EOM in the same optical temperature measurement setup. Optical temperature sensors based on the temperature-dependence spectrum or polarization state variations in various sensing transducers have been successfully developed [12–14]. An optical birefringence-based temperature sensor, using a single-crystal sapphire, was proposed for high temperature measurement in [12]. In this measurement, a lithium niobate plate was used as a sensing transducer based on a thermally-induced birefringence change [15]. The temperature information was encoded in the phase delays between two orthogonal polarizations when the incident light passed through the LN plate under the ambient temperature changes. The results show that the similar measurement sensitivities are about 25 deg/°C for both the ZIEMO and EOM at a 632.8 nm wavelength. We found that the EOM cannot be used for modulating the stable HLS at a 532 nm wavelength due to the essential photorefractive effects. However, the HLS modulated by the ZIEOM is stable enough for optical temperature measurements with higher measurement sensitivity of 30 deg/°C.

## 2. Experimental Setup

**Figure 1.**
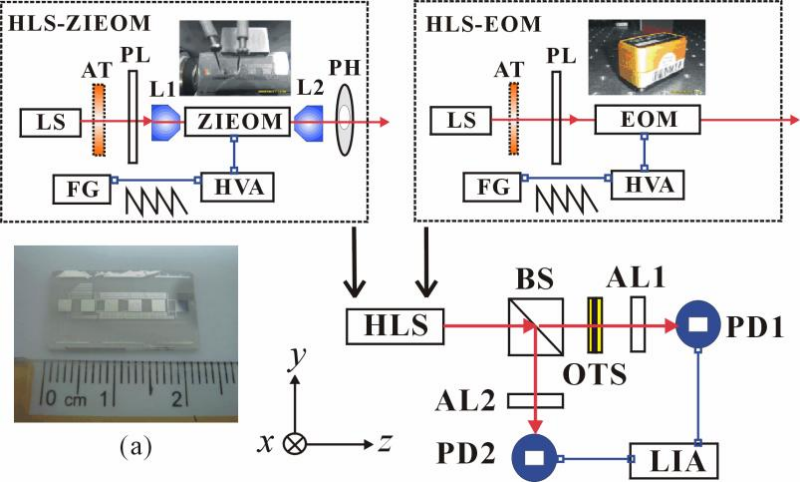
The schematic diagram of the measurement setup: LS, laser light source; ZIEOM, Zn-indiffused electro-optic modulator (photo shown in Inset (a)); EOM, buck-type electro-optic modulator; FG, function generator; HVA, high voltage amplifier; HLS, heterodyne light source; AT, attenuator; PL, polarizer; PH, pinhole; AL, analyzer; L, objective lens; BS, beam splitter; PD, photodetector; OTS, optical temperature sensor; LIA, lock-in amplifier.

The schematic diagram of the measurement setup is shown in Figure 1. The ZIEOM (photo shown in inset (a)) was made by using a thermally Zn-indiffused waveguide in the *x*-cut/*z*-propagating LN substrate. The detailed fabrication process of the device has been reported in [11]. The width of the waveguide was 4 μm. The length and gap-width of the parallel electrodes were 10 mm and 24 μm, respectively. A He-Ne laser (632.8 nm) and a diode-pumped solid-state laser (532 nm) were used for the measurements. The optimum waveguide fabrications can obtain a single guided mode for both orthogonal polarizations at wavelengths of 532 nm and 632.8 nm. The input powers were controlled by using an attenuator (AT). The linear polarization at +45° with respect to the *x*-axis of the laboratory coordinates was obtained after passing through a polarizer (PL). The incident light was coupled into the ZIEOM through an objective lens (L1). The output guided light from the ZIEOM was collected by another objective lens (L2). A pinhole (PH) was adopted to block the scattering light after the L2. In the EOM (Model 4002, New Focus, Inc.) measurement setup, the L1, L2 and PH can be removed. A beam splitter (BS) was used to spatially divide the incident light into two paths. The transmitted light (sensing path) passing through the analyzer (AL1) was received by a photodetector (PD1). An optical temperature sensor (OTS) was placed in the sensing path. The reflected light (reference path) passing through the analyzer (AL2) was received by the photodetector (PD2). The transmission angles of both analyzers AL1 and AL2 are perpendicular to that of the PL. The final transmitted and reflected signals are sinusoidal curves under the applied saw-tooth voltages from a function generator (FG) amplified by a high voltage amplifier (HVA).

The normalized output intensities of the transmitted and reflected signals (*I*_T_ and *I*_R_) can be expressed by:

(1) $$I_{T}=\frac{1}{2}(1-\text{cos}(2\pi \mathrm{ft}+\phi_{\mathrm{PR}}(t)+\phi_{\mathrm{temp}}(T)))$$

(2) $$I_{R}=\frac{1}{2}(1-\text{cos}(2\pi \mathrm{ft}+\phi_{\mathrm{PR}}(t)+\phi_{\mathrm{BS}}))$$

where *f* is the frequency of the applied voltages, *ϕ*_BS_ is the stable path-dependent phase difference for the transmitted and reflected lights due to the different propagation conditions in the BS, *ϕ*_PR_(t) is a time-varying phase delay between orthogonal polarizations in the ZIEOM and EOM due to photorefractive effects, and *ϕ*_temp_(t) is a temperature dependent phase variation when the incident light passes through the OTS. To compare the phase stabilities of the HLS generated by different modulators (without the OTS), we further observed the time-varying phase variations detected by the lock-in amplifier (Model 7225, Signal Recovery, Inc.) under different throughput powers (measuring in front of the BS) at a modulation frequency of 100 kHz.

## 3. Optical Temperature Sensing Principle

A thin (1 mm) lithium niobate plate was used as the optical temperature sensor based on a thermally-induced birefringent change. The plate was immersed in hot water in a glass cell. The incident light propagates perpendicularly to the *c*-axis of the LN plate which is parallel to the *x*-axis of the laboratory coordinate. The water temperature was cooled naturally and monitored by using a *K*-type thermocouple for a period of 10 minutes. The temperature dependent phase retardations between the two orthogonal polarizations of the incident light are represented by:

(3) $$\begin{array}{ll} \phi_{\mathrm{temp}}(T) & =\frac{2\pi }{\lambda }\cdot l(T)\cdot \left|n_{e}(T)-n_{o}(T)\right|\\ & =\frac{2\pi }{\lambda }\cdot l(25{}^{\circ}C)\cdot [1+\alpha \cdot (T-25{}^{\circ}C)]\cdot \left|n_{e}(T)-n_{o}(T)\right| \end{array}$$

where the temperature value is denoted by *T*(°C), *n_e_*(*T*) and *n_o_*(*T*) are the temperature-dependence extraordinary and ordinary refractive indices, respectively, λ is a wavelength of the incident light, *l*(T) is a temperature-dependence thickness of the LN plate, *l*(25 °C) is the thickness of the LN plate at room temperature, and *α* is a thermal expansion coefficient (*α* = 1.5 × 10^−5^/°C) [16]. The temperature dependent phase retardations can be measured by using the setup as shown in Figure 1. According to the relationship between phase variation and the temperature change, the measurement sensitivity is defined as:

(4) $$\begin{array}{ll} S & =\;\left|d(\phi_{\mathrm{temp}}(T))/\mathrm{dT}\right|\\ & =\frac{2\pi }{\lambda }\cdot l(25{}^{\circ}C)\cdot [\alpha \cdot \left|n_{e}(T)-n_{o}(T)\right|+(1+\alpha \cdot (T-25{}^{\circ}C))\cdot \left|d(n_{e}(T)-n_{o}(T))/\mathrm{dT}\right|]\\ & \approx \frac{2\pi }{\lambda }\cdot l(25{}^{\circ}C)\cdot [\alpha \cdot \left|n_{e}(T)-n_{o}(T)\right|+\left|d(n_{e}(T)-n_{o}(T))\mathrm{dT}\right|] \end{array}$$

where *α* (*T* – 25 °C) << 1 can be ignored. The measurement sensitivity can be obtained by calculating the absolute slopes of the raw data for phase variations *versus* temperatures. Based on the calculated measurement sensitivity, we also can obtain a thermal-optic birefringence coefficient for the LN crystal which is expressed as:

(5) $$\left|d(n_{e}(T)-n_{o}(T))/\mathrm{dT}\right|\approx [S\cdot \lambda /l(25{}^{\circ}C)\cdot 2\pi ]-\alpha \cdot \left|n_{e}(T)-n_{o}(T)\right|$$

## 4. Results and Discussion

**Figure 2.**
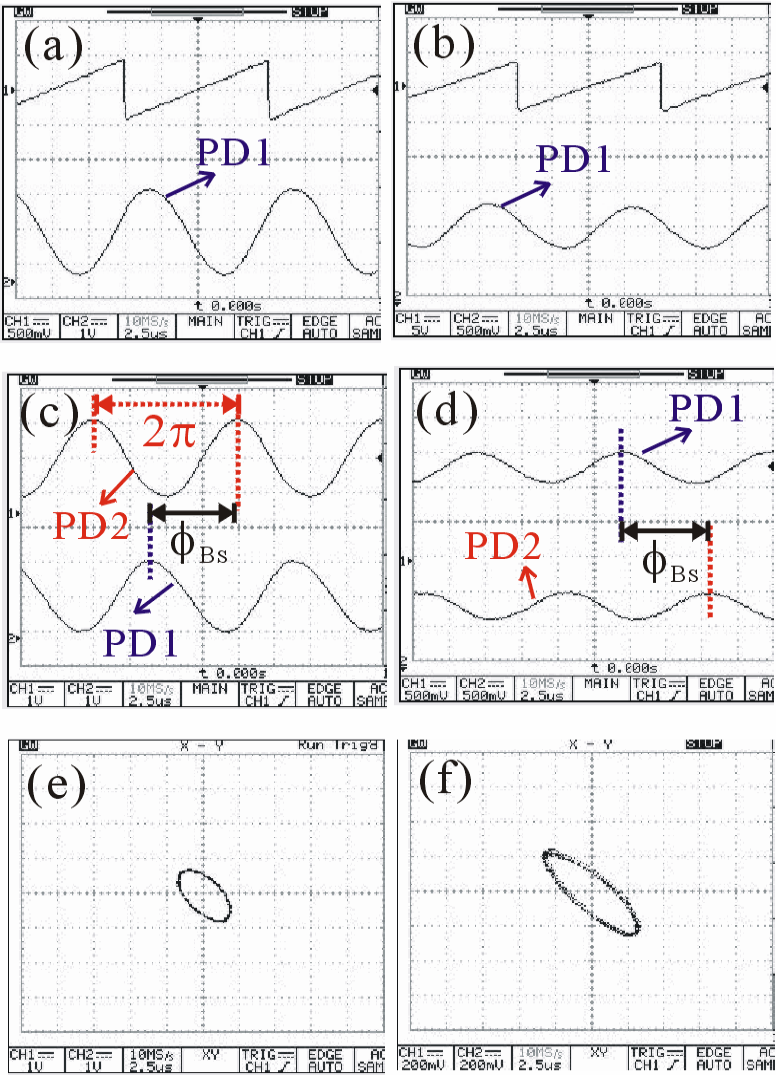
The measured oscilloscope traces at 632.8 nm: **(a)** the applied saw-tooth voltage and optical response curve for ZIEOM; **(b)** the applied saw-tooth voltage and optical response curve for EOM; **(c)** the dual optical response curves for ZIEOM; **(d)** the dual optical response curves for EOM; **(e)** the Lissajous curve for ZIEOM; and **(f)** the Lissajous curve for EOM.

Figure 2 presents the measured oscilloscope traces for the ZIEOM and EOM under the same modulation frequency of 100 kHz at 632.8 nm. The applied saw-tooth voltages and the optical response curves are shown in Figures 2(a,b) for the ZIEOM and EOM, respectively. The measured saw-tooth voltages are from the FG. Actually, the applied voltages are amplified by 40-fold in the ZIEOM and EOM via the HVA. The best optimized peak voltages to obtain the ideal sinusoidal waveforms of the output optical signals are 18 V and 140 V for ZIEOM and EOM, respectively. The smaller driving voltages of the ZIEOM are due to the narrower gap width (24 μm) of the parallel electrodes compared with that in the EOM (≈2 mm). According to Figures 2(c,d), the beam splitter will cause a phase shift *ϕ*_BS_ between the two different light paths. The calculated values are about 1.5π for the heterodyne light sources from both EOM and ZIEOM. Besides, the Lissajous curves can also represent the static phase shift from the beam splitter, as shown in Figures 2(e,f). Similar elliptic Lissajous curves are obtained for both modulators.

**Figure 3.**
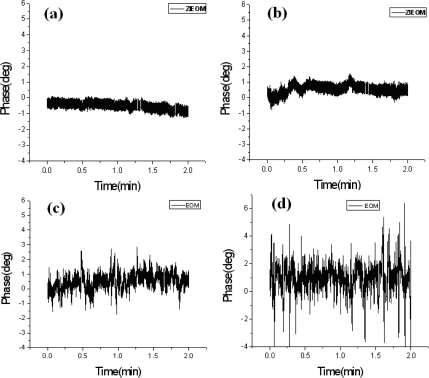
Phase variation *versus* time for the ZIEOM and EOM under different throughput powers at 632.8 nm: **(a)** 25 μW for the ZIEOM; **(b)** 15 μW for the ZIEOM; **(c)** 25 μW for the EOM; and **(d)** 15 μW for the EOM.

To measure the time-varying phase variations, we adopted the LIA with a sampling rate of 200 Hz to collect 24,000 data within two minutes. First, the OTS (shown in Figure 1) was removed to evaluate the inherent stability of the system. The measured results of the phase stabilities depending on the throughput powers for the ZIEOM and the EOM are shown in Figure 3. According to the results, the phase variations of the ZIEOM are more stable compared with those of the EOM at 632.8 nm. Then, we further studied the capability of the ZIEOM used for the HLS at 532 nm.

**Figure 4.**
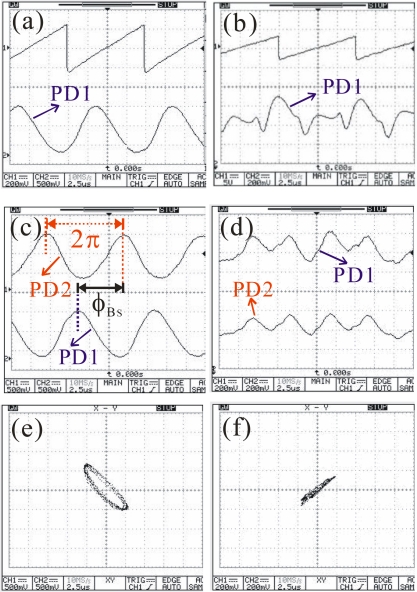
Measured oscilloscope traces at 532 nm: **(a)** the applied saw-tooth voltage and optical response curve for ZIEOM; **(b)** the applied saw-tooth voltage and optical response curve for EOM; **(c)** dual optical response curves for the ZIEOM; **(d)** dual optical response curves for the EOM; **(e)** the Lissajous curve for the ZIEOM; and **(f)** the Lissajous curve for the EOM.

Figure 4 shows the measured oscilloscope traces at 532 nm. The applied saw-tooth voltages and the optical response curves are shown in Figures 4(a,b) for the ZIEOM and EOM, respectively. The applied peak voltages are 12 V and 120 V for the ZIEOM and EOM, respectively. The optical powers are the same at 25 μW measured in front of the BS for both modulators. The EOM shows that the optical response traces are disorderly, due to the essential impacts of photorefractive effects. The dual optical response curves from the ZIEOM are stable, as shown in Figure 4(c). The calculated *ϕ*_BS_ is about 1.5π. However, the distorted waveforms of the dual channel signals from the EOM are impossible to calculate *ϕ*_BS_, as shown in Figure 4(d). The Lissajous curve of the ZIEOM is shown in Figure 4(e). The elliptic pattern is also similar to Figures 2(e,f). The Lissajous curve of the EOM is unstable. One of the random Lissajous curves is shown in Figure 4(f). These corrupted dual signals will cause a failed phase locking in the LIA. Therefore, it is still difficult to obtain reliable phase information for the EOM, even when using a lower power of 6 μW. In contrast, the optical response curves of the ZIEOM are still sinusoidal-like waveforms, which can be locked easily via the LIA.

**Figure 5.**
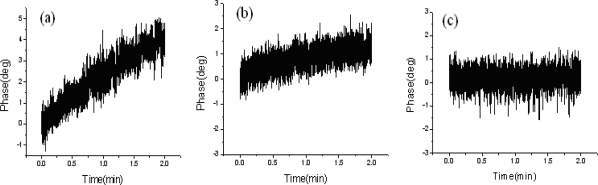
Phase variation of different throughput powers for the ZIEOM at 532 nm: **(a)** 25 μW; **(b)** 15 μW; and **(c)** 6 μW.

The phase variations of the ZIEOM under different throughput powers are shown in Figure 5. The gradual increasing biases of phase variations are obvious at 25 μW due to slight photorefractive effects. In the case of a throughput power of 15 μW, the phase variations of the ZIEOM are much more stable compared with those of the EOM at 632.8 nm, as shown in Figure 3(d). When the power is reduced to 6 μW, the biases of phase variations are near stable. We believe that the ZIEOM can be used for generating a stable HLS at 532 nm under the prompt throughput powers.

**Figure 6.**
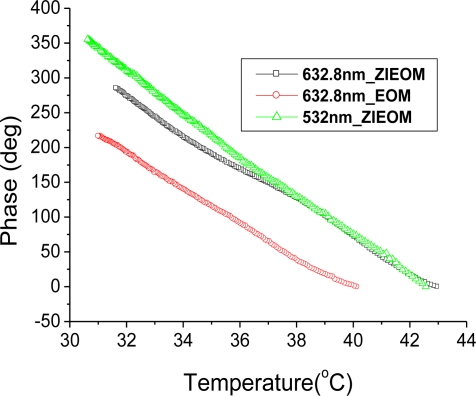
Optical temperature measurement results.

To demonstrate the optical metrology capabilities of the HLS generated by the ZIEOM, the temperature measurement characteristics are compared with the HLS generated by the EOM under similar test conditions. Further, the 532 nm-HLS from the ZIEOM is used for optical temperature measurements. Figure 6 gives the phase variations *versus* temperature changes for them. According to Equation (4), the absolute slopes of the linear fit of the measured data can be defined as the measurement sensitivity. In the incident wavelength of 632.8 nm, the measurement sensitivities are almost the same values of 25 deg/°C for both the ZIEOM and EOM. Based on Equation (5), we expect |*n_e_*(*T*) – *n_o_*(*T*)| ≈ 8 × 10^−2^ in the temperature changes, and the value of the product *α* |*n_e_*(*T*) – *n_o_*(*t*)| to be around 1.2 × 10^−6^ /°C The calculated thermal-optic birefringence coefficients of the LN plate are about 4.2 × 10^−5^. This value is close to the measured value (≈3.9 × 10^−5^/°C) from the literature [17]. This means that the ZIEOM can obtain the same measurement sensitivity as the EOM. However, the applied voltage of the ZIEOM is much less than that of the EOM. According to previous discussions, as shown in Figure 4, only the ZIEOM can generate stable HLS at a wavelength of 532 nm. The measurement sensitivity is improved up to 30 deg/°C under the throughput power of 6 μW. Without the OTS in the sensing path, the average phase variation of the measurement system is about ±1 deg, as shown in Figure 5(c), and the measurement resolution of 0.07 °C is achievable. When the OTS is put in the sensing path and the water is at room temperature in the measurement period of 10 minutes, the long-term stability measurements show that the average phase variation is about ±1.25 deg, and the measurement resolution is about 0.08 °C. To monitor rapid temperature changes, the response time of the optical temperature sensor is another important consideration. The response time of the sensing element is dependent on the time required to reach thermal equilibrium with the measuring environment [12]. The heat transfer between the sensing element and the surrounding environment is mainly dependent on the physical properties of the sensing element, such as thermal conductivity, specific heat, and geometrical size. Because the LN plate is immersed in hot water, convection and conduction behaviors are the main mechanisms for heat transfer. Although we do not observe the sensing response time of the proposed LN plate in this paper, we believe that the smaller size of the LN plate is better to reduce the response time according to discussions presented in [12]. A large dynamic sensing range is also important. In the LIA-based detections, the limited dynamic range of phase measurements is from −180 deg to +180 deg. The measurement sensitivity of the 1 mm-thick LN plate is 25 deg/°C for a sensing wavelength of 632.8 nm. The measured phase is ambiguous when the temperature changes are over 15 °C. In principle, the dynamic range can be increased up to 150 °C by using a thinner LN plate of 0.1 mm.

## 5. Conclusions

In summary, we have experimentally evaluated the ZIEOM with a low applied voltage used for optical heterodyne interferometry. To provide heterodyne light sources at a wavelength of 632.8 nm, the optimal peak values of the applied saw-tooth voltages are 18 V and 140 V for ZIEOM and EOM, respectively. Unlike the light propagating perpendicularly to the *c*-axis of the LN crystal in the EOM, the *z*-propagating ZIEOM shows more stable phase operations due to the lesser photorefractive effects. Besides, we also proved that the ZIEOM can be used for optical temperature measurements. The results show that the measurement sensitivities are about 25 and 30 deg /°C for the wavelengths of 632.8 and 532 nm, respectively. The best measurement resolution is about 0.07 °C at the sensing wavelength of 532 nm. Moreover, the integrated ZIEOM is potentially packaged easily with visible light sources to provide a compact module for an optical instrument.
